# STAR-VITAL, a Four Year Comprehensive Workplace Health Promotion Program: Study Design

**DOI:** 10.3390/ijerph19105854

**Published:** 2022-05-11

**Authors:** Klemen Širok, Mojca Stubelj, Matej Voglar, Denisa Manojlović, Darinka Radoja, Suzana Laporšek, Matija Vodopivec, Ana Arzenšek, Natalija Rozman, Mirna Macur, Katja Pesjak, Simona Perčič

**Affiliations:** 1 Faculty of Health Sciences, University of Primorska, 6310 Izola, Slovenia; klemen.sirok@fvz.upr.si (K.Š.); mojca.stubelj@fvz.upr.si (M.S.); matej.voglar@fvz.upr.si (M.V.); denisa.manojlovic@fvz.upr.si (D.M.); darinka.radoja@fvz.upr.si (D.R.); 2 Faculty of Management, University of Primorska, 6310 Izola, Slovenia; suzana.laporsek@fm-kp.si (S.L.); matija.vodopivec@fm-kp.si (M.V.); ana.arzensek@fm-kp.si (A.A.); 3 National Institute of Public Health Ljubljana, 1000 Ljubljana, Slovenia; natalija.rozman@nijz.si; 4 Angela Boškin Faculty of Health Care, 4270 Jesenice, Slovenia; mmacur@fzab.si (M.M.); kpesjak@fzab.si (K.P.)

**Keywords:** workplace health promotion program (WHPP), interventions, customer relationship management (CRM), participation, effectiveness

## Abstract

**Background: **Premature death, chronic disease, and productivity loss can be reduced with the help of programs that promote a healthy lifestyle. Workplace health promotion programs have been shown to be an efficient way of improving employee health. These can also benefit employers by improving retention, reducing worker turnover, and lowering healthcare costs. In Slovenia, a workplace health promotion program called “STAR-VITAL—Joint Measures for the Vitality of Older Workers” targeting small- and medium-sized enterprises has been ongoing since September 2017. We hypothesize that this workplace health promotion program will yield long-term health changes for the included employees and employers. **Methods/Design:** The manuscript presents a workplace health promotion program design that introduces some novel approaches and solutions to workplace health promotion program implementation. It also introduces a measurement of their effects that address the problem of low participation rates and the effectiveness of workplace health promotion programs, as follows: (1) the multifaceted and individualised approach to implementation, (2) customer relationship management (CRM) -based interaction management with program participants, and (3) impact evaluation based on employee health and labour market data observing both intermediate outcomes and the final outcomes based on national micro administrative data. **Discussion:** Although the novel approaches introduced with the STAR-VITAL program proved to be effective during the COVID-19 pandemic, they deserve the attention of scholars and practitioners. Further research is called for to further explore the potential of CRM in health promotion contexts, the effectiveness of multifaceted and individualised workplace health promotion program interventions, and micro administrative data-based impact evaluations. **Conclusions:** The STAR-VITAL program introduces several new approaches addressing the problem of low participation rates and the effectiveness of WHPPs. Further research is called for to discover and explore the potential of those novel approaches.

## 1. Introduction

One of the central roles in managing health and chronic disease prevention is implementing a workplace health promotion program (WHPP) [1]. WHPPs are employer-based training programs with the ultimate aim of improving the organizational health of participating employers, with the support of (certified) trainers. An emphasis is placed on strategies to reduce the chronic disease and injury risk of employees and improve overall worker productivity [2]. WHPPs combine the efforts of employers, employees, and society to improve the mental and physical health and well-being of people at work [2]. A World Health Organization report from 2018 [3] clearly shows that chronic conditions like cardiovascular diseases, cancer, chronic respiratory diseases, type II diabetes, and obesity are the leading causes of death and disability worldwide. Health promotion activities (increasing physical activity, healthy eating, stress management, sleep hygiene, and healthy relationships) are important in preventing non-communicable, lifestyle-related diseases, and can increase personal resilience and improve overall health [4]. Promoting both healthier nutrition and more physical activity is considered to be the most important defence in combatting obesity and diabetes and preventing cardiovascular diseases [5]. Studies have shown that chronic stress is associated with weight gain, abdominal adiposity, and obesity. Stress can encourage individuals to eat more sugar, fat, and salt, and is associated with binge eating habits [6]. Stress is also associated with smoking, alcohol consumption, physical inactivity, and sleep disturbance [7]. Furthermore, sleep deprivation may lead to obesity [8], while sleep disturbances (too much or too little sleep) are associated with a greater risk of all-cause mortality [9]. Reduced vigilance, cognitive impairments in memory and executive function, decreased memory processing, and deprived immune system function are also associated with getting too little sleep [10,11]. Moreover, the quality of a person’s interpersonal relationships has a profound influence on their health. For example, social support is associated with less smoking, alcohol consumption, and obesity, and a lower frequency of symptoms like fatigue, headaches, depression, and anxiety in the workplace [12,13,14]. It has been documented that WHPPs (promoting healthy nutrition, adequate levels of physical activity, sleep hygiene, healthy relationships at work, etc.) can prevent at least 70% of chronic noncommunicable diseases [15].

The population in Slovenia is clearly aging, and in the coming years, more older employees are expected to suffer from chronic noncommunicable diseases. This will increase absentee rates due to the functional limitations of employees and present an increased financial burden to employers. Aside from absentee rates, the decline in health and poor lifestyle habits of older employees also decreases their productivity [16]. Another reason for implementing WHPPs is that employees often lack the time, equipment, or other facilities, and the support and expertise needed for such activities after work and in the home environment [17]. As a consequence, WHPPs can offer advantages to employees, employers, and society as a whole [18,19]. However, many previous studies postulated that the design of such programs must be carefully considered, noting that online platforms about healthy lifestyles are not enough on their own, but that other activities related to healthy lifestyle habits must also be implemented [20]. Education should be incorporated in any WHPP for a sufficient period of time (at least three years, along with other promotional programs) to allow individuals to build knowledge and values, reframe their habits and work practices, and develop strategies for a healthy lifestyle [20].

The number of WHPPs has increased significantly over the past 30 years, and many studies show that such programs are effective and well-received in organizations [20,21,22,23]. However, WHPPs are only successful if they integrate and involve all stakeholders. Moreover, they should also be integrated into all areas of the organization and include the development of a comprehensive prevention policy with a strategy to improve employee health [24].

Despite the many positive effects of WHPPs, as reflected in increased productivity, reduced sick leave, and healthier lifestyles among participating individuals, actual participation rates are critically low [25,26]. Studies to date suggest that participation rates in such programs are often below 50% [27,28] and that WHPPs tend to attract healthier employees rather than those in need [29,30]. This greatly reduces the potential benefits and cost-effectiveness of these programs [25,26] and decreases the chance of positive changes occurring in maintaining employee vitality [31].

In Slovenia, a WHPP program called “STAR-VITAL—Joint Measures for the Vitality of Older Workers” has been ongoing since September 2017. This five year program is co-financed by the Ministry of Labour, Family, Social Affairs and Equal Opportunities of the Republic of Slovenia and the European Union’s Social Fund. STAR-VITAL is led by independent professionals from much-respected institutions (National Institute of Public Health, Faculties, the Chamber of Craft and Small Business of Slovenia) in our country, and their work does not depend on direct payments by companies. The STAR-VITAL program was publicly funded (companies did not pay for the services).

The project is dedicated to promoting healthy lifestyle habits and preventing and managing chronic diseases in the workplace. While comparable workplace health promotion programs focus mainly on large multinational companies, STAR-VITAL aims to improve the working environment of small- and medium-sized enterprises (SMEs) in Western Slovenia. Based on the detailed needs analysis and expectations of small- and medium-sized enterprises, the STAR-VITAL program raises employee awareness and encourages both management and employees to exercise more, eat healthier, and develop sleep hygiene. STAR-VITAL is therefore designed to better individualize chosen strategies [32] in comparison with health promotion programs in multinational companies. The program teaches the participants stress management techniques and educates them to help develop better interpersonal relationships and intergenerational cooperation, which was often neglected in previous studies [32]. Through mentoring, counselling, coaching, interactive workshops, and incentives, the project aims to reduce sick leave so that employees can work healthier for longer. Within the project, employers work together with the project team to create healthier workplaces and strengthen the sense of responsibility employees have for their own health. Under mentorship, the firms’ managers learn about the legal and tax aspects of health promotion, create a workplace health promotion plan, develop better use of the experience and knowledge of their older employees, and increase employee satisfaction and performance. The STAR-VITAL program also aims to empower individual organizations to continue promoting healthy lifestyle habits even after the project has ended.

The aim of this manuscript is to present a WHPP design that introduces some novel approaches and solutions when it comes to the implementation of WHPP programs and the measurement of their effects. This article presents the STAR-VITAL program study design with an emphasis on the following elements developed to address the problem of low participation rates and effectiveness of WHPPs: (1) the multifaceted approach to implementation, (2) customer relationship management (CRM) -based interaction management with participants, and (3) impact evaluation based on employee health and labour market data observing both intermediate outcomes (i.e., change in body weight or risk factors of the target population due to participation in the interventions) and the final outcomes (i.e., effects on absences from work due to illness) based on national micro administrative data.

## 2. Methods/Design

### 2.1. Study Design

The STAR-VITAL program has been designed and implemented by four partners in the project: two faculties from the field of health sciences and the National Institute of Public Health in Slovenia, covering different aspects of employee health, and the Chamber of Craft and Small Business of Slovenia, which provided links between the medical experts and firms. The project team consists of experts from different backgrounds: a nutritionist, a kinesiologist, physiotherapists, psychologists, bio-psychologists, public health and management experts, and experts in statistical and econometric methods.

The STAR-VITAL program has been conducted in several phases. First, based on a review of best practices in the field of active and healthy ageing at work, both in Slovenia and abroad, and a detailed analysis of the situation in Slovenia, the target groups and work-related health issues, as well as the general determinants of absenteeism and the retirement postponement, were identified. Secondly, a multi-dimensional model of active and healthy ageing was developed, building upon the WHO Model of Healthy Workplaces [33], including a toolbox of WHPP interventions. In the third phase of the project, the eligible organizations were approached to start with the implementation of interventions. The interventions started in June 2019 and are still ongoing, and the fourth phase of the project includes an evaluation of these. This evaluation consists of two parts: (i) evaluation of the intervention effects on the level of both individual employee and firm, and (ii) evaluation of the intervention effects on absences from work due to illness and the effects on retirement.

### 2.2. Recruitment of the Participants

#### 2.2.1. Participation in the Program

Participation in the program was voluntary and free of charge for eligible firms, as well as for individual employees. The STAR-VITAL program was initially intended for workers aged 45 or more. Nevertheless, the firm-level analysis of the situation regarding healthy lifestyle habits at the workplace and work-related health risks showed that these issues were equally important for younger workers. Therefore, all employees in the participating firms can, on a voluntary basis, be included in the interventions.

The STAR-VITAL program focuses on SMEs in the western cohesion region of Slovenia, with a small number of larger firms also included, as required by the financing body. At first, the project team prepared a list of eligible firms in the region. Those firms were then contacted by a member of the team by phone, who briefly explained the purpose and aims of the project. In-person meetings between project team representatives and the firm’s management were organized at those companies that expressed interest in co-operation. If the firm decided to participate in the project, the project team organized a promotional workshop about the STAR-VITAL program and the importance of taking care of one’s own health in the work environment for all employees in the firm. Everyone who expressed a willingness to participate in the project was asked to give written informed consent for participation and to complete the screening questionnaire. This questionnaire detects the exposure to work-related health risk factors, current lifestyle habits (nutrition, stress exposure, physical activity, and sleep hygiene), work-related information (communication, work climate, and existing WHPP activities), and intention to change, and collects relevant socio-demographic data. The screening questionnaire was initially offered in paper-and-pencil format, but this was later supplemented with an online questionnaire via an e-platform (presented below), whereby each employee and/or employer is assigned an ID to anonymize the data. Each employee who completes the screening questionnaire is automatically registered as a new user in the e-platform. Screening questionnaires are then analysed for each individual firm and aggregated findings presented to the employer. From June 2019 a total of 1061 employees completed the screening questionnaire.

Before the start of the project’s activities, all participants were informed that their anonymity and confidentiality would be protected, based on the European Union General Data Protection Regulation (GDPR) rules. Employee data are kept confidential and are not disclosed to the employer. Participants can withdraw their consent for participation voluntarily and at any time. There are no specific risks associated with participating in the program other than those associated with the adoption of an active lifestyle. The study protocol was also approved by the Slovenian National Medical Ethics Committee (No. 0120–345/2018/9).

#### 2.2.2. Selection of Interventions

Based on the findings from the screening questionnaire and in-depth discussions with the employer, a firm-specific intervention plan is prepared. In addition, screening questionnaire data are used to segment the employees according to existing or potential health risks so that they can be later assigned appropriate interventions (i.e., lower back interventions for sedentary workers). Program interventions are carried out by a team of professionals from two faculties of health care (all with PhDs in their respective fields): the faculty of management (HR specialist and two economists, all PhDs) and the National Institute of Public Health (two public health specialists).

We have to point out that the STAR-VITAL project has an important adherence with the SMEs included The professional team of the STAR-VITAL is in continuous contact with an individual SME. All members of the team are available at any time for employers either on telephone or face to face (using mostly Zoom in the last couple of years). We are continuously in contact with all SMEs for discussion about their progress and interventions selection, which are suitable for them in the future according to the up to date results. Workshops are tailor-made, enabling employees to individually express their wishes and questions. Live conferences were prepared and were very successful as different SMEs discussed the problems and good practices with each other (so that communication was not only encouraged intra-company, but also inter-company). Personal contact between the professional team, employers, and employees proved to be of vital importance for the success of interventions.

After the interventions, the employees are asked to fill out the screening questionnaire again, which enables an analysis of changes in their behaviour and makes it possible to obtain information on their degree of satisfaction with different interventions.

### 2.3. Project Interventions

The STAR-VITAL program offers SMEs a range of WHPP interventions that are gathered under the STAR-VITAL platform umbrella. In the project, we work with managers, employees, and health promoters. WHPP content is tailored to the needs and expectations of businesses, whereby each participating firm chooses interventions on a voluntary basis and they are based upon the previous expert analysis of the data in the screening questionnaire. Altogether, eight different interventions and/or tools, which are presented in the Table 1, were created by the professional team involved in the project.

Some interventions are targeted at the individual employee or company level, while group interventions make use of sharing knowledge and experience, as well as group dynamics. Initially, most interventions were intended to be performed on-site (face-to-face), but due to the COVID-19 pandemic, these were also adapted for synchronous online implementation using the Zoom application, along with the existing e-platform functionalities that can be delivered via PCs or mobile devices using responsive web design. The project’s website is currently in the public domain, while the e-platform and STAR-VITAL Wiki page are only available to the participating companies.

#### 2.3.1. e-Platform

The e-platform is a platform solution specifically focusing on individuals—an employee, health promoter, or manager—based on a customer relations management (CRM) system. This platform provides more than 250 landing pages with videos, educational content, templates, and interactive questionnaires, along with active lifestyle challenges, whereby participants’ activities are scored, and in certain activities, also rewarded. Within the e-platform we encourage companies and employees to change their health-related habits in the work environment with targeted activity programs, or in CRM terms, health promotion campaigns. Activity programs/promotion campaigns are defined by a matrix formed by target groups (managers, employees, and health promoters) and areas of health promotion (HR practices supporting WHPP and an ageing workforce, physical activity and ergonomics, nutrition, stress, sleep, communication and leadership, intergenerational dialogue, absenteeism, legal and tax aspects of occupational health promotion, and work–life balance). The e-platform also enables the use of the short message service (SMS) as a secondary communication channel. The e-platform has several functions:

- a screening questionnaire with a participant registration function for the e-platform itself (already described above),
- individual activity programs/campaigns,
- distribution of general WHPP information to companies,
- generation and distribution of interactive content(s), like screening questionnaires and active lifestyle challenges,
- user activity monitoring, and
- in-depth user analytics and content evaluation.

Each activity program is treated as a communication/marketing campaign that is distributed by the CRM system and is designed as follows: Individual employees (depending on their position—manager, employee, or health promoter—and identified health risk factors) receive via their personal email one unit of content per week (or other pre-set time interval). This unit of content can be either informative (i.e., the importance of lower sugar intake) or instructional (i.e., a video with a set of exercises for the lower back). Within one campaign, the employee receives 10 to 15 content units over a period of two to three months. Prepared content units follow contemporary web content and writing style guidelines and rely on a considerable amount of video content.

The CRM-based e-platform also enables monitoring user activities and distribution of automatic reminders in case of a participant’s inactivity. This is done by the integrated email tracking (i.e., monitoring of email delivery) function. Each email sent through e-platform is monitored with regard to the number of users to whom the email was sent, the number of users to whom the email was unsuccessfully delivered (i.e., it remained unopened, for whatever reason), and the number of users who read the email. In addition, the e-platform identifies the most frequently read emails, as well as the user ratings of individual landing pages. Combining those two data sources on the reach of the content allows the in-depth data analysis of participants’ activity and the quality and usefulness of the content. In order to additionally encourage participation, STAR-VITAL active lifestyle challenges are also offered to companies whereby employees are rewarded with token gifts, depending on how many challenges from the physical activity, nutrition, sleep, and interpersonal relations sections they complete.

Through the e-platform, firms also receive recent news from the field of healthy lifestyle habits, regional sports events, and (during peaks of the epidemic) COVID-19 prevention measures for different work environments. This general information is thematically designed in such a way that companies can easily integrate it into their internal communication channels. Typical topics include nationwide or regional health promotion events, recommendations for dealing with the COVID-19 situation, seasonally appropriate food, and so on.

#### 2.3.2. Workshops

Simultaneously with the e-platform campaigns that also set the implementation pace, interactive workshops for all nine areas of health promotion mentioned above are provided for different target groups in companies. These can take place on-site (face-to-face) or via video conferencing systems that enable individual and/or group work (Zoom). During 2021, the STAR-VITAL expert team conducted 30 workshops (physical activity, eating habits, sleep hygiene, etc.), with the participation of 319 individuals across the included companies. Additionally, we included 31 new companies in the program and started a new campaign promoting physical activity in the workplace environment. Accordingly, 19 companies received exercise aids and were regularly followed by a physiotherapist (live, by telephone or video call).

#### 2.3.3. Coaching and Mentoring

In order to provide active assistance in the implementation of WHPP measures, the workshops are supplemented with coaching and mentoring available to the firms’ management and health promotors. The ‘train the trainer’ activities are provided on-site (face-to-face) or online with the aim of empowering those who are critical for the successful implementation of WHPP measures. Coaching and mentoring focus on the development of the various tactics and skills required to introduce changes in organizations and sustain the introduced activities following the conclusion of the STAR-VITAL program. Managers have the opportunity to talk actively with our experts and to the trainers from other firms. Workshops, coaching, and mentoring are all aimed at developing the STAR-VITAL community and stimulating the exchange of good WHPP practices. The advantage to reach high adherence with included SMEs may be that Slovenia is very small. The advantage of the STAR-VITAL team is its familiarity with Slovenian-specific culture, which enables the implementation of WHPP interventions in Slovenian SMEs. The advantage may also be that most of the SMEs of Western Slovenia know each other, and each of them has its own reputation—both are well known to our professional team.

#### 2.3.4. Support in Creating a Favourable Work Environment

The STAR-VITAL program also offers an in-depth analysis of the problematic aspects of the work environment that expose workers to health risks. On the basis of in-depth ergonomic or nutritional analyses, experts also prepare recommendations for improvement. For instance, the nutritionist and public health expert advise the firms or local meal providers on healthier menus. Physiotherapists and kinesiologists introduce the use of various ergonomic tools, such as ergonomic keyboards, computer mice, adjustable workstations, and so on. The firms are also provided with exercise equipment to facilitate the implementation of active breaks during working hours. In order to provide employees with continuous support, specifically tailored exercises are sent to the individuals’ personal email addresses and updated on a weekly basis. Not only were theoretical instructions prepared, but practical solutions in individual SMEs were also prepared when visiting individual SMEs. Final decisions on interventions for a particular SME were made together with management of the SME, who would then incorporate them in their company’s vision and strategy.

#### 2.3.5. STAR-VITAL Wiki Page

The STAR-VITAL Wiki page provides firm managers with descriptions of over 160 WHPP measures that are presented using a unified structure: a general description of the focal WHPP measure, accompanied by a description of an implementation case, with emphasis on the details that were key for successful implementation. The descriptions of the measures follow the contemporary web content and writing style guidelines, with special attention paid to usability, with user-friendly forms of information search available (search engine, content tree structure, and tag-based filtering).

#### 2.3.6. STAR-VITAL Website

The home page of the project (https://www.star-vital.si/, accessed on 3 December 2021) presents the project to the general public, attracts firms’ interest, and raises general awareness. An important element of the website is a repository of articles from the fields of WHPP and healthy lifestyle that were written for a general audience. On the website, employees are encouraged to take part in STAR-VITAL (Ljubljana, Slovenia) 15 min active breaks, which are broadcasted live on weekdays via videoconference. There is also a recording of the workout from the previous day that is available for those who missed the live stream.

#### 2.3.7. Conferences

Annual conferences are organized within the project. These conferences address different audiences: the academic community, practitioners, managers of SMEs, and the broader public. At the beginning of the project, the conferences were more focused on raising awareness among managers, but later, this was replaced with presentations of different STAR-VITAL tools and methods. In the concluding phase of the project, the conference topics focus more on presentations of good practices and challenges that were encountered during the implementation phase. Live conferences were prepared and were very successful as different SMEs discussed the problems and good practices with each other (so the communication was not only encouraged intra-company, but also inter-company). Personal contact between the professional team, employers, and employees proved to be of vital importance for the success of interventions. All papers and contributions to the annual STAR-VITAL conferences are published on the project’s home page.

#### 2.3.8. Media Appearances

Throughout the project, different media channels have been used to disseminate results and information on different topics concerning lifestyle and health (e.g., best nutritional ingredients, cardiovascular health, diabetes, obesity, etc.) relevant to the public. In a nationwide, monthly magazine called *Obrtnik/Podjetnik*, published by the Chamber of Craft and Small Business of Slovenia, a special section is dedicated to the STAR-VITAL program. Collaboration with the national TV was also established to provide TV coverage for STAR-VITAL program activities and promote workplace health and longer careers.

### 2.4. Statistical Analyses

The evaluation of the interventions of the STAR-VITAL program will be conducted in two phases: measuring intermediate outcomes (i.e., changes in the behaviour of the target population due to inclusion in the interventions) and final outcomes (i.e., effects on absences from work due to illness and the effects on retirement).

In the first phase, we will analyse whether the above-described interventions improved the intermediate outcomes (i.e., working environment, nutritional habits, strategies of coping with stress, and sleep hygiene). The analysis will be based on data gathered with questionnaires, which were filled out by all employees before the start of the interventions and at the end of the project. The analysis will include a comparison of responses before and after the interventions for all participants and will be disaggregated by gender, age group, characteristics of workplace, type of firm, sector of activity, etc. In addition, a multinominal regression model will be used to assess differences in habits and attitudes. The dependent variable will be distance travelled (difference in observed aspects of intermediate outcomes), whereas independent variables will include personal characteristics (gender, age, and education), occupation, characteristics of a firm, where the employee is employed, and a dummy variable for observation period.

The second phase of evaluation will focus on the effects of the project’s interventions on final outcomes (absences from work due to illness and the effect on the type of retirement and age at retirement). The estimation of the effects will be based on the difference-in-differences method, comparing experimental and control groups before and after interventions. The experimental group will be composed of all employees included in the STAR-VITAL program, whereas the control group will include comparable individuals from other Slovenian firms that were not included in the project. The control group will be formed using propensity score matching. The difference-in-differences method enables the accurate identification of the effects of interventions, as it eliminates the effects of other interfering factors (e.g., changes in the structure of the considered samples due to ageing and environmental factors), while simpler methods (e.g., simple comparisons of indicators) cannot. The aim of the analyses will be to estimate the “Intent to Treat Effects” and not the “Average Treatment Effect on the Treated”, as not all workers were included in all the interventions. The difference between these two analyses is due to the fact that despite being employed by those firms included in the STAR-VITAL program, the employees did not participate in all the interventions that were offered. As these people are likely to be systematically different from the others—they are chosen non-randomly—the effect of the measures on the persons involved cannot be considered as a causal effect of the inclusion. Estimates of “intended involvement” thus do not reflect the direct effects of the intervention on the participating workers, but rather the combined effect of the firm being involved in the project and encouraging workers to participate in the intervention.

The analyses of the second phase of the evaluation will be based on extremely rich administrative data that provide both labour market and health information on the entire population of Slovenia from 2015 onwards, with employment spells being linked to their employers. The dataset is built from the following sources, all having nationwide coverage:

#### 2.4.1. Labour-Market Related Databases:

*Work history database*, including information on the starting and ending dates of an employment spell, the type of appointment, occupation, regular number of hours of work, employer identification code, and personal characteristics (gender, age, and education). Data were obtained from the Statistical Office of the Republic of Slovenia.

*Database on registered unemployment*, including the starting and ending dates of each unemployment spell, destination of exit, and personal and family characteristics. The source of data is the Employment Service of Slovenia.

*Database on persons who retired from January 2015 onwards*, including data on date and type of retirement. Data are provided by the Pension and Disability Institute of Slovenia.

*Data on business performance of firms*, including data from financial statements and data on the characteristics of firms.

#### 2.4.2. Health and Health-Related Databases:

Data on temporary and permanent absences from work due to illness, injury, care, escort, and other reasons, including data on the cause and duration of absence, were found in these databases. The causes of absence are coded according to ICD-10. The data are provided by the National Institute of Public Health.

#### 2.4.3. STAR-VITAL Data: List of Firms Included in the STAR-VITAL Interventions

The individual- and firm-level data from various sources will be merged on the basis of a personal identification number (PIN) and through the employer identification code, while employment spells will also be linked to accounting and other data on the current employers. Access to data—and the bulk of the empirical analysis—will take place in the Statistical Office’s “safe room”. Before allowing access to researchers, the data will be anonymized, and thus the information on the related person’s name, surname, address of residence, and PIN will be removed.

## 3. Discussion

The STAR-VITAL program introduces several new approaches to addressing the multifaceted problem of low participation rates and the effectiveness of WHPPs that deserve the attention of scholars and practitioners. It is interesting that despite many positive effects perceived from other WHPPs, participation rates are critically low [25,26]. WHPPs have positive effects on employee behaviours (in terms of encouraging regular physical activity, healthy eating, stress management, sleep hygiene, and healthy relationships) which increase personal resiliency and improve health [19,29,30,31,32,33,34,35,36,37,38,39,40,41,42,43,44]. The potential strength of the STAR-VITAL program is that it addresses multiple variables in the work environment (work-related health risks) and behavioural habits (physical activity and eating habits) of employees in SMEs. Using an individualized strategy and a wide range of interventions, higher efficiency and participation rates are expected. According to Robroek et al. [27], a higher participation rate can be obtained when an incentive is offered, and the intervention consists of multiple components or is aimed at multiple behaviours and variables.

According to the literature, the main barriers to participation are lack of time [45], lack of awareness and misconceptions about workplace health [46], and privacy factors and moral concerns about employer’s interference in employees ’personal lifestyle decisions [47]. Lack of time, as one of the main and most common barriers to participation in the WHPP, was taken into consideration when preparing the program and individual interventions. Companies were offered precisely what they were found to be needing according to the screening questionnaire. The implementation of the program in a particular company was individualized, which means that it was adapted to the known needs of employees and was targeted at problematic areas according to the screening questionnaire. Interventions were designed to be implemented during working hours and to achieve the greatest possible impact in the shortest possible time. Lack of awareness and misconceptions about health in the workplace have been addressed through a variety of awareness-raising activities: articles on website, campaigns on the e-platform, media appearances, and participation at conferences. Participants were guaranteed anonymity and confidentiality through the informed consent form. Participation was completely voluntary and could be terminated at any time without giving a reason. The program is designed holistically, which means that it tries to cover as many areas related to providing better working conditions and strengthening the individual in caring for their health in the workplace as possible. Although the common belief seems to be that intrinsic motivators are the best driver for sustained change, some small extrinsic incentive rewards in form of practical prizes (water bottles, soft balls, pedometers, etc.) were used to drive employee engagement in some of the activities offered by the program. In addition, due to the national and European financial contribution, the program was completely free of charge for all parties involved.

The implementation of the project showed that mixed communication channels and individualized multifaceted interventions increase implementation flexibility and improve the project outreach. Due to the COVID-19 pandemic and lockdown periods, it was impossible to conduct interventions in a face-to-face format. In order not to cease ongoing and planned activities, these were moved to an online environment. This was found to be an additional benefit to users, as it was a further choice for the SMEs. The companies themselves thus chose the method of workshop implementation (live or online), which meant gaining a greater number of participants. Although the project initially targeted older workers aged 45 and up with weaker information technology literacy levels, the project’s e-health interventions have proven to be a successful solution and promising way to promote healthy employee behaviours during the COVID-19 situation.

A special feature of the STAR-VITAL program is the use of CRM in the e-platform, which enables regular sending of information to, and activity monitoring of, the employees. At the same time, automatic reminders are distributed to inactive participants. To the best of our knowledge, this is the first WHPP that uses a CRM in its online platform. CRMs are generally used in competitive consumer markets for building and maintaining good and long-term relationships with customers [48]. The reason for the introduction of a CRM in our WHPP was so that we could have regular contact with the employees and gain more insights into their activities and needs. Further research is called for to discover and explore the potential of using a CRM in health promotion contexts.

Another special feature of the STAR-VITAL program is the planned evaluation based on micro administrative data using a dataset derived from reliable sources provided by the Statistical Office of the Republic of Slovenia and the National Institute of Public Health for the entire population of Slovenia from 2015 onwards. To the best of our knowledge, this will be the first WHPP to analyse employee health and labour market data in the manner described. Most interventions measure intermediate outcomes (i.e., change in body weight or risk factors of the target population due to inclusion in the interventions), such as those described by Anderson et al. [49], but in our intervention, we will also look at the final outcomes (i.e., effects on absences from work due to illness).

All of the STAR-VITAL interventions were conducted in SMEs. According to Tremblay et al. [50], occupational health and safety is poorer in SMEs than in large corporations due to a lack of knowledge, time, motivation, resources, and skills. SMEs have been recognized as work environments that are hard to reach and difficult to change, while at the same time dealing with a complex range of needs in order to manage the health and well-being of their employees [51,52]. As a result, SMEs may not be in a position either to invest time and effort in the offered interventions or access the available services. This poses a significant challenge for us and also for both employers and managers in SMEs.

Some limitations of this work should also be noted. The data collection method was based on filling out questionnaires, and most of the variables of interest, such as the level of physical activity and eating habits, in both the current program and its variations, come from these self-reported answers. The objective measures obtained, for example, from accelerometer data, heart rate monitors, blood tests, and pedometers, could be used to identify the changes that were taking place at the individual level in further work. Another issue is that the SMEs that enrol in the project are self-selected, and the control group will include firms that do not want to be included in the project. There are fears that there will be undetected differences between the experimental and control groups (e.g., in the management/leadership style of running the company and in the importance that companies give to a culture of participation) that will affect the project impact assessment (probably in terms of overestimation, as companies are likely to be more in favour of the project measures, and therefore the proposed measures will “fall on fertile ground”). One proposal to overcome this is that among those companies that apply to join the project, we randomly select one group (experimental) and we have the remaining companies as a control group. In order to examine whether unobserved characteristics play an important role, the proposed evaluation will employ a difference-in-differences approach, which can account for time invariant firm-level heterogeneity.

## 4. Conclusions

The STAR-VITAL program introduces several new approaches addressing the problem of low participation rates and effectiveness of WHPPs. First, the program addresses multiple variables in the work environment (work-related health risks) and behavioural habits (i.e., physical activity, eating habits, and sleep hygiene), applying individualized implementation strategies utilizing a wide range of interventions. Coupled with the use of the mixed communication channels and increased implementation flexibility, improved project outreach and higher participation rates are expected. A special feature of the STAR-VITAL program is the use of a CRM in the e-platform, which enables treating and managing the WHPP interventions as marketing campaigns. Another novel approach introduced by the STAR-VITAL program is the planned evaluation based on national micro administrative data, estimating the effects focusing on the final outcomes (i.e., effects on absences from work due to illness) through the difference-in-differences method, comparing experimental and control groups before and after interventions. Further research is called for to discover and explore the potential of those novel approaches.

## Figures and Tables

**Table 1:** Projects interventions and activities.

Interventions/Methods/Tools	Target Group	Level of Intervention	Target Areas/Activities
e-platform	All employees in SMEs	Managers	Individual activity programs/campaigns; distribution of general WHPP information to companies; generation and distribution of interactive content(s), such as active lifestyle challenges; and activity monitoring.
		Employees	
		Health promoters	
Workshops	All employees in SMEs	Managers	Nutrition, stress management, physical activity, ergonomics, sleep hygiene, communication, work climate, absenteeism, and implementation of WHPP workplace health promotion program activities.
		Employees	
		Health promoters	
Coaching and mentoring	All managers and health promoters in SMEs	Managers	Exchange of good WHPP practices and development of the various tactics and skills required to introduce changes in organizations.
		Health promoters	
Support in creating a favourable work environment	All managers and health promoters in SMEs	Managers	Advice to the firms or local meal providers on healthier menus and advice for keyboards, computer mice, adjustable workstations, and exercise equipment provision for active breaks during working hours.
		Health promoters	
STAR-VITAL Wiki page	All employees in SMEs	Managers	Over 160 WHPP measures with key information for successful implementation.
		Employees	
		Health promoters	
STAR-VITAL website	All employees in SMEs	Managers	The 15 min active breaks, which are broadcasted live on weekdays
		Employees	
		Health promoters	
Conferences	Academic community, practitioners, managers of SMEs, and the broader public	Managers	Contents raising awareness among managers, presentations of different STAR-VITAL tools and methods, and presentations of good practices and challenges.
		Employees	
		Health promoters	
Media appearances	Academic community, practitioners, managers of SMEs, and the broader public	Managers	Information on monthly magazine called *Obrtnik/Podjetnik* and participation on the national TV.
		Employees	
		Health promoters	

Legend: WHPP: workplace health promotion program; SME: small- and medium-sized enterprises; TV: television.

## Data Availability

Not applicable—the results will be communicated to the participants and to other relevant groups via publications (open access will be prioritized) and presentations, including webinars.

## Abbreviations

CRM	customer relationship management
GDPR	General Data Protection Regulation
ICD-10	International Statistical Classification of Diseases and Related Health Problems
PIN	personal identification number
SME	small- and medium-sized enterprise
SMS	short message service
WHPP	workplace health promotion program
